# Editorial: Clinical Application of Artificial Intelligence in Emergency and Critical Care Medicine, Volume I

**DOI:** 10.3389/fmed.2021.809478

**Published:** 2021-12-06

**Authors:** Zhongheng Zhang, Nan Liu, Qinghe Meng, Longxiang Su

**Affiliations:** ^1^Department of Emergency Medicine, Sir Run Run Shaw Hospital, Zhejiang University School of Medicine, Hangzhou, China; ^2^Programme in Health Services and Systems Research, Duke-National University of Singapore Medical School, Singapore, Singapore; ^3^Department of Surgery, State University of New York Upstate Medical University, Syracuse, NY, United States; ^4^State Key Laboratory of Complex Severe and Rare Diseases, Department of Critical Care Medicine, Peking Union Medical College Hospital, Chinese Academy of Medical Science and Peking Union Medical College, Beijing, China

**Keywords:** prediction, artificial intelligence, critical Care, emergency medicine, precise medicine

Analytics based on artificial intelligence (AI) has greatly advanced a variety of scientific research fields such as natural language processing, imaging classification and signal processing (1). Clinical research is also revolutionized by the development of artificial intelligence (2), and conventional research paradigm is being supplemented by the new technology. Conventional treatment strategy based on evidence-based medicine typically exploits the average treatment effect in a population to dictate medical decision making (3). However, it is well-known that a patient population is usually heterogeneous that one size does not fit all. In other words, although a treatment strategy is reported to be beneficial for the overall population, it might be harmful for a subgroup of patients. Thus, the idea of individualized treatment is proposed to address the problem of differential treatment effects in a heterogeneous population. Patients in emergency and critical care setting are usually heterogeneous and the clinical condition changes rapidly (4, 5), which highlights the importance of early risk stratification and individualized treatment.

Artificial intelligence can be applied in three aspects in the emergency and critical care setting. These three aspects have been well-captured in this Research Topic entitled “Clinical Application of Artificial Intelligence in Emergency and Critical Care Medicine, Volume I,” which has been successfully launched in Frontiers in Medicine. First, several studies developed prediction models for risk stratification in the critical care setting. Different clinical risks are defined in a variety of study populations such as mortality prediction in surgical ICU patients, risk of blood transfusion in liver transplantation, and risk of coagulopathy in sepsis. Collectively, these studies exploited the supervised learning algorithm to train a prediction model (6). The clinical events of interest/labels must be unambiguously defined. Misclassification in the database will cause model instability or inaccuracy for the prediction in future samples (7). The second category of study is to disentangle heterogeneous population into more homogenous subgroups by using unsupervised machine learning algorithms (8). The algorithms differ from the supervised learning methods in that they do not require the samples being labeled in advance. Instead, they exploit the features to classify samples into separable subgroups/subtypes. The subgroups of patients can have prognostic and predictive enrichment. Prognostic enrichment indicates different subgroups have different risk of clinical outcome events, whereas the predictive enrichment indicates that different subgroups can have different responses to a particular intervention. In this collection of articles, Zhang et al. explored the subphenotyes of acute respiratory distress syndrome and found that Rosuvastatin has differential treatment effect across these subphenotypes. Chen et al. developed a novel clinical classification system for SARS-CoV-2 Pneumonia, which showed prognostic enrichment for mortality outcome. They further developed a parsimonious class membership prediction model for the ease of clinical utility. The third type of clinical scenario is to employ reinforcement learning algorithm to dictate treatment regimen in sequential manner (9, 10). This methodology is not used in the current collection of articles. The key idea underlying this application is that the treatment strategy should be tailored sequentially according to the changes of patient's status. The interactions between treatment action, patient state, and reward are formalized in a dynamic process, so as to maximize the final outcome reward. Alternatively, the dynamic treatment regime (DTR) model adapts the idea of reinforcement learning to estimate a sequence of decision rules, one per stage of intervention, that dictate how to individualize treatments to patients based on evolving treatment and covariate history. DTR relaxes the model complexity and are more acceptable to the field of medical epidemiology. This model has been utilized in critical care setting to tailor fluid resuscitation in sepsis and ventilation strategy in acute respiratory failure (10, 11).

Since the advances in machine learning algorithms have greatly revolutionized the industry, the technology can surely influence how we treat patients in the emergency and critical care setting. However, the application of AI in clinical practice is still in its infancy and requires more research efforts. Several key aspects that hinder the utility of AI models in clinical practice include but not limit to the quality of training datasets, institutional idiosyncrasy, and model overfitting (12). That is why some models show good performance in the training dataset but perform poorly in new samples. The model might learn something specific to an institution/hospital, but not the underlying true pathophysiological processes. The second issue relates to the model interpretability. Although AI models can improve prediction accuracy in some situations, a notorious drawback of these models are their black box nature prohibiting easy interpretation of the predicted outcome (13, 14). Physicians are less likely to adopt a recommendation made by the machine while the underlying pathophysiology is unknown or uninterpretable. Due to the importance and the potential impact of artificial intelligence on the emergency and critical care setting, we launch a second volume of the Research Topic. We welcome more studies to address the above-mentioned problems in applying ML in clinical practice. Successful settlement of these issues will hopefully transform more research models into real clinical practice.

## Author Contributions

ZZ conceived the idea. QM, LS, and NL critically reviewed the manuscript and revised the paper. All authors contributed to the article and approved the submitted version.

## Funding

The study was funded by the Health Science and Technology Plan of Zhejiang Province (2021KY745), Yilu “Gexin” – Fluid Therapy Research Fund Project (YLGX-ZZ-2020,005).

## Conflict of Interest

The authors declare that the research was conducted in the absence of any commercial or financial relationships that could be construed as a potential conflict of interest.

## Publisher's Note

All claims expressed in this article are solely those of the authors and do not necessarily represent those of their affiliated organizations, or those of the publisher, the editors and the reviewers. Any product that may be evaluated in this article, or claim that may be made by its manufacturer, is not guaranteed or endorsed by the publisher.
